# Supplementary material for: The genome sequence of the pond louse, Asellus aquaticus (Linnaeus, 1758)
Source: Wellcome Open Res. 2025 Jun 2;10:291. [Version 1] doi: 10.12688/wellcomeopenres.24299.1 (PMC12800620; doi:10.12688/wellcomeopenres.24299.1)
Supplement: Peer review history

# Reviewer report: Reviewer response for version 1

**DOI:** 10.21956/wellcomeopenres.26797.r143369

**Published:** 2026-01-13

**Authors:** Martin Schwentner

**Affiliations:** 1 Natural History Museum Vienna, Vienna, Austria

The author, Jessica Thomas, provides the first chromosome-scale geome assembly of the freshwater isopod species A. aquaticus, which is of evolutionary as well as ecological importance. The results are highly relevant, well presented and the underlying methods and protocols clearly explained and presented. All relevant data is shown and a part from two minor questions I am very satisfied with the manuscript and have nothing to add or criticize

 1. is there a citation for the ASCC pipeline?

 2. The analyses relies on  large number of scripts (I guess including many own pipelines etc). It would be great if collection of scripts and commands used herein were provided as well (maybe they are and I just missed them)

Are sufficient details of methods and materials provided to allow replication by others?

Yes

Is the rationale for creating the dataset(s) clearly described?

Yes

Are the datasets clearly presented in a useable and accessible format?

Yes

Are the protocols appropriate and is the work technically sound?

Yes

Reviewer Expertise:

phylogenomics

I confirm that I have read this submission and believe that I have an appropriate level of expertise to confirm that it is of an acceptable scientific standard.

# Reviewer report: Reviewer response for version 1

**DOI:** 10.21956/wellcomeopenres.26797.r125959

**Published:** 2025-11-03

**Authors:** Tara Cronin

**Affiliations:** 1 Clemson university, Clemson, USA

**Article – **The genome sequence of the pond louse, *Asellus aquaticus *(Linnaeus, 1758)

**Summary – **In this study, the author presents the first documentation of the genome assembly of the pond louse, *Asellus aquaticus*. It is part of the Darwin Tree of Life project which aims to sequence all eukaryotic species in the Atlantic Archipelago of Britain and Ireland. The authors produced a genome sequence of 1,977.80 Mb, with the majority of the assembly being scaffolded into 8 chromosomal pseudomolecules. Additionally, the authors assembled the mitochondrial genome of this species. The authors measured the QV and k-mer completeness, along with BUSCO scores for the assembly. These measures resulting in a QV of 53.3, k-mer recovery of 97.95%, and a BUSCO score of 88.4%

**Comments related to questions-**

1. Is the rationale for creating the dataset clearly described?
  The dataset created is part of a much larger project, The Darwin Tree of Life project, which aims to sequence all name eukaryotic species in the Atlantic Archipelago of Britain and Ireland. Additionally, the emergence of several isolated subterranean populations that have evolved cave-associated traits has made this organism a model taxon to study parallel adaptations. This genomic resource will allow for characterization of genomic changes driving evolution of these subterranean populations. The authors describe both the project and the need/importance of this genome clearly.
2. Are the protocols appropriate and is the work technically sound?

The authors used appropriate protocols and the work appears to be technically sound. The protocols are clearly outlined for the entire Darwin Tree of Life project, allowing for all genomes sequenced for this project to have the same protocol. Numerous other publications have already used this protocol adding to the support of its appropriateness.

1. Are sufficient details of methods and materials provided to allow replication by others?

Yes sufficient details are provided to allow for replication. The authors do a good job describing their methods in the paper, and provide access to all their protocols so that others may replicate this study with ease.

1. Are the datasets clearly presented in a useable and accessible format?
  The dataset is archived in repositories online allowing for others to access all the raw data and assembly data. Additionally, the figures and tables provide a good presentation of the data for readers to get a good understanding of what is in the repositories.

**Minor Revisions –**

 Line 48, Left column – provide the suborder of the woodlouse in paraentheses.

 Line 51, Left column – Write out *Asellus aquaticus *rather than using the abbreviated form, as it is at the beginning of a sentence.

 Line 53, Left column – provide a citation for the sentence about the distribution of this species.

 Line 57, Left column – Change *Asellus aquaticus* to *A. aquaticus*, as you have already written out the full name and it is not the start of the sentence.

 Line 72, Left column - Change *Asellus aquaticus* to *A. aquaticus*, as you have already written out the full name and it is not the start of the sentence.

 Line 82, Left column – change Gb to gigabases (Gb) as it is the first time the abbreviation is use and should be defined.

 Line 84, Left column – change Mb to megabases (Mb) as it is the first time the abbreviation is use and should be defined.

 Line 88, Left column – change 56 to 56x.

 Line 89, Left column – remove the comma between reads and and.

 Line 56, Right column – provide a link or name of the repositories you are referring to so that readers can quickly find it.

 Line 157, Left column – Change *Asellus aquaticus* to *A. aquaticus*, as you have already written out the full name and it is not the start of the sentence.

 Line 140, Right column – there is a paraentheses missing after the ToLID number.

 Line 154, Right column – It is not clear that this is a standardized protocol the way the sentence currently is written. Mentioning that this protocol is standardized would help the reader know that more detailed methods can be found in that protocol.

 Line 158, Right column – state what the relevant DNA barcode region is for this sample to help with clarity for the reader.

 Line 207, Left column – to keep a consistent style with the rest of the paper, and to increase the ease and ability of the reader to replicate your methods, provide the manufacturer name and headquarters location for the PowerMasher II tissue disruptor.

 Line 207, Left column – Generally starting sentences with abbreviations should be avoided, change HMW to High Molecular Weight (HMW) as it is the start of the sentence and the first time this term is used.

 Line 210, Left column - to keep a consistent style with the rest of the paper, and to increase the ease and ability of the reader to replicate your methods, provide the manufacturer name and headquarters location for the Megaruptor 3 system.

 Line 212, Left column - to keep a consistent style with the rest of the paper, and to increase the ease and ability of the reader to replicate your methods, provide the manufacturer name and headquarters location for the AMPure PB beads.

 Line 198, Right column - to keep a consistent style with the rest of the paper, and to increase the ease and ability of the reader to replicate your methods, provide the manufacturer name and headquarters location for the Nanodrop spectrophotometer, Qubit Fluorometer, and the Qubit dsDNA High Sensitivity Assay kit.

 Line 201, Right column - to keep a consistent style with the rest of the paper, and to increase the ease and ability of the reader to replicate your methods, provide the manufacturer name and headquarters location for the FemtoPulse system.

 Line 210, Right column - to keep a consistent style with the rest of the paper, and to increase the ease and ability of the reader to replicate your methods, provide the manufacturer name and headquarters location for the Qubit RNA Broad-Range Assay kit.

 Line 211, Right column - to keep a consistent style with the rest of the paper, and to increase the ease and ability of the reader to replicate your methods, provide the manufacturer name and headquarters location for the Agilent RNA 6000 Pico Kit and Eukaryotic Total RNA assay.

 Line 247, Left column - to keep a consistent style with the rest of the paper, and to increase the ease and ability of the reader to replicate your methods, provide the headquarters location for the Arima-HiC v2 kit.

 Line 252, Left column - to keep a consistent style with the rest of the paper, and to increase the ease and ability of the reader to replicate your methods, provide the manufacturer name and headquarters location for the Diagnocine Power Masher II.

 Line 254, Left column - to keep a consistent style with the rest of the paper, and to increase the ease and ability of the reader to replicate your methods, provide the manufacturer name and headquarters location for the SPRIselect beads.

 Line 256, Left column - to keep a consistent style with the rest of the paper, and to increase the ease and ability of the reader to replicate your methods, provide the headquarters location for the Qubit Flurometer v4.0.

 Line 245, Right column - to keep a consistent style with the rest of the paper, and to increase the ease and ability of the reader to replicate your methods, provide the manufacturer name and headquarters location for the Qubit HS assay Kit.

 Line 246, Right column - to keep a consistent style with the rest of the paper, and to increase the ease and ability of the reader to replicate your methods, provide the manufacturer name and headquarters location for the Arima-HiC v2 QC beads.

 Line 255, Right column - to keep a consistent style with the rest of the paper, and to increase the ease and ability of the reader to replicate your methods, provide the headquarters location for the SMRTbell Prep Kit 3.0.

 Line 283, Left column - to keep a consistent style with the rest of the paper, and to increase the ease and ability of the reader to replicate your methods, provide the headquarters location for the AMPure PB beads.

 Line 284, Left column - to keep a consistent style with the rest of the paper, and to increase the ease and ability of the reader to replicate your methods, provide the headquarters location for the Qubit Flurometer v4.0 and the Qubit 1X dsDNA HS assay kit.

 Line 287, Left column - to keep a consistent style with the rest of the paper, and to increase the ease and ability of the reader to replicate your methods, provide the headquarters location for the Agilent Femto Pulse and gDNA 55kb BAC analysis kit.

 Line 291, Left column - to keep a consistent style with the rest of the paper, and to increase the ease and ability of the reader to replicate your methods, provide the headquarters location for the Sequel IIe system and Revio.

 Line 280, Right column - to keep a consistent style with the rest of the paper, and to increase the ease and ability of the reader to replicate your methods, provide the manufacturer name and headquarters location for the SMRTbell beads.

 Line 292, Right column - to keep a consistent style with the rest of the paper, and to increase the ease and ability of the reader to replicate your methods, provide the manufacturer name and headquarters location for the Covaris E220 sonicator.

 Line 294, Right column - to keep a consistent style with the rest of the paper, and to increase the ease and ability of the reader to replicate your methods, provide the manufacturer name and headquarters location for the Arima-HiC v2 Enrichment beads.

 Line 332, Left column - - to keep a consistent style with the rest of the paper, and to increase the ease and ability of the reader to replicate your methods, provide the headquarters location for the prep kit.

 Line 336, Left column - to keep a consistent style with the rest of the paper, and to increase the ease and ability of the reader to replicate your methods, provide the manufacturer name and headquarters location for the KAPA HiFI HotStart mix.

 Line 337, Left column - - to keep a consistent style with the rest of the paper, and to increase the ease and ability of the reader to replicate your methods, provide the headquarters location for the adapters.

 Line 343, Left column - - to keep a consistent style with the rest of the paper, and to increase the ease and ability of the reader to replicate your methods, provide the headquarters location for Biotium.

 Line 344, Left column - to keep a consistent style with the rest of the paper, and to increase the ease and ability of the reader to replicate your methods, provide the manufacturer name and headquarters location for the Illumina NovaSeq.

 Line 349, Left column - to keep a consistent style with the rest of the paper, and to increase the ease and ability of the reader to replicate your methods, provide the headquarters location for the RNA library Prep Kit.

 Line 340, Right column – Provide a citation for the ASCC pipeline.

**Major Revisions – N/A**

Are sufficient details of methods and materials provided to allow replication by others?

Yes

Is the rationale for creating the dataset(s) clearly described?

Yes

Are the datasets clearly presented in a useable and accessible format?

Yes

Are the protocols appropriate and is the work technically sound?

Yes

Reviewer Expertise:

Mitochondrial genomics, behavioral ecology, invertebrate biology, physiology, parasitology

I confirm that I have read this submission and believe that I have an appropriate level of expertise to confirm that it is of an acceptable scientific standard.
